# Measuring Body Composition in Individuals with Intellectual Disability: A Scoping Review

**DOI:** 10.1155/2013/628428

**Published:** 2013-05-15

**Authors:** Amanda Faith Casey

**Affiliations:** Department of Human Kinetics, St. Francis Xavier University, P.O. Box 5000, Antigonish, NS, Canada B2G 2W5

## Abstract

*Background*. Research shows obesity to be more prevalent amongst individuals with intellectual disability (ID) making correct measurement of body composition crucial. This study reviewed the validity and reliability of methods used for assessing body composition in individuals with ID. *Methods*. Authors conducted electronic searches through PubMed (1990 to present) and PsycINFO (1990 to present) and assessed relevant articles independently based on scoping review guidelines. Reviewers included primary research related to the validity and reliability of body composition measures on individuals with ID. *Results*. Searches identified six articles assessing body composition methods used on individuals with ID including body mass index (BMI), skinfold thickness, bioelectrical impedance analysis (BIA), waist circumference, tibia length, and anthropometric girth measurements. BMI and waist circumference appear suitable measures but skinfold thickness measurements may not be advisable due to participants' noncompliance resulting in a lack of precision and inaccurate results. *Conclusions*. The current literature contains too few well-conducted studies to determine the precision and validity of body composition measures on individuals with ID. There may be a need to devise further regression equations that apply to individuals with specific types of ID in order to increase the reliability and validity of body composition measurements.

## 1. Introduction

Individuals with intellectual disability (ID) are at increased risk for obesity and extreme obesity [1], which contribute to numerous cardiovascular, pulmonary, and metabolic diseases [2, 3]. More specifically, research documents physiological mechanisms that associate total and regional body fat with insulin resistance, glucose metabolism, serum lipid concentrations, and blood pressure [4]. 

Recommended by the World Health Organization [5], body mass index (BMI) and waist circumference are used frequently to measure obesity across different populations. Yet, there remains a question as to the extent these methods accurately reflect body composition or fat distribution in individuals with ID who often display unique anthropometry compared to individuals without disabilities [6]. 

Several alternative solutions are available to scientists and practitioners seeking to assess obesity in individuals with different types of ID. Laboratory or “reference” methods, such as air displacement plethysmography (ADP), hydrostatic weighing, and dual-energy X-ray absorptiometry (DXA), are conducted often with reliable results on diverse populations [7] even if their expense and lack of portability sometimes limit their use in community-based settings. Field methods including skinfold thickness and bioelectrical impedance analysis (BIA) also offer practical and more cost-effective alternatives but, unlike the high-precision laboratory methods, the accuracy of these methods remains dependent largely upon specific regression equations that should be selected on the basis of a participant's age, gender, ethnicity as well as physical activity, and body fat levels. Research demonstrates that such equations should be limited only to the type of population in which they have been validated otherwise there is an increased risk that they may underestimate or overestimate body fat levels [8–11]. 

It is essential to know which body composition methods are accurate and feasible for determining health status in individuals with ID. However, no study to our knowledge has reviewed measures used on this population despite the growing efforts being made to combat obesity through health promotion initiatives [12]. Therefore, the purpose of this study is to review the reliability and validity of methods used for assessing body composition in individuals with ID.

## 2. Aim and Methods of Review

Researchers carried out a scoping review relating to the validity and reliability of body composition measures in individuals with ID. A scoping review offers a primary evaluation of the range of the available literature on a particular topic [13] and is especially pertinent in disability and health research where there remains a lack of uniformity in the study design and measurement. The author followed the framework of Arksey and O'Malley [14] who underscored five key phases when conducting a scoping review: (i) identifying the research question; (ii) identifying relevant studies; (iii) study selection; (iv) charting the data; and (v) collating, summarizing, and reporting the results. 

The scoping review addressed the following questions. (1) What measurement tools have researchers used when assessing body composition in individuals with ID? (2) What are the validity and/or reliability of these methods according to the empirical literature? In order to address these questions,researchers sourced journal articles from PubMED (1990–2012) and PsycINFO (1990–2012) and retrieved articles using the keywords “intellectual disability” and “mental retardation” in conjunction with “body composition,” “body fat,” “anthropometry,” and “obesity”. Reviewers excluded review articles but examined their reference lists to highlight relevant articles. Reviewers included primary research related to the validity and reliability of body composition measures on individuals with ID. For validation purposes, the following measures are included: coefficient of determination (*r* ^2^); coefficient of correlation (*r*); root mean square error (RMSE) or standard error of estimation (SEE); bias (mean difference between the alternative and the criterion method), and the agreement (usually assessed by the Bland-Altman method) represented by the upper and lower of the 95% confidence intervals of the bias (mean difference ±2 standard deviations). The following parameters are used for assessing reliability: intraclass coefficient of correlation (ICC, also reported for validation purposes); Cohen's kappa; coefficient of variation (CV); and technical error of measurement (TEM). For inclusion, studies had to (i) feature a population with any kind of ID [15]; (ii) assess body composition; and (iii) evaluate the validity and/or reliability of body composition measures. No study was excluded based on the methodology but the scope was limited to studies written in English and published in peer-reviewed journals between 1990 and 2012. 

**Figure 1:**
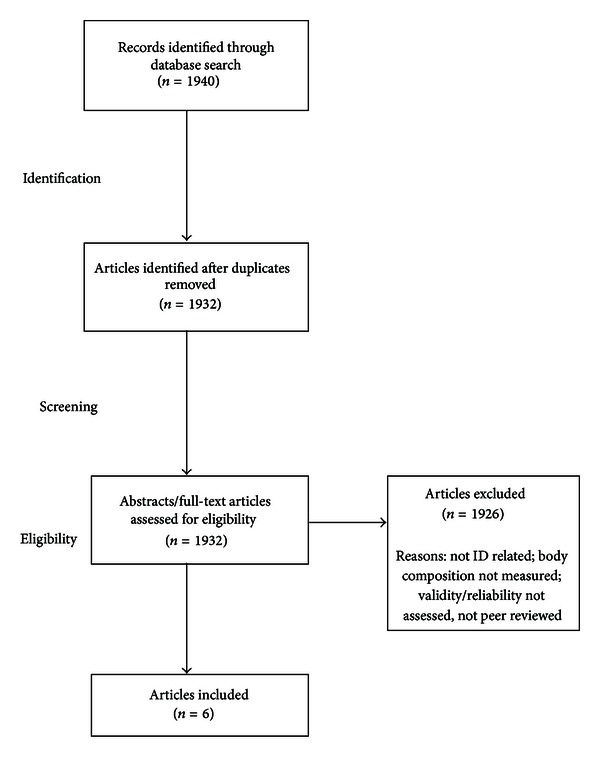
Scoping review of the literature on body composition measures for individuals with intellectual disability.

The main author, along with a peer, screened titles, abstracts, and results for inclusion independently. When disagreement occurred concerning inclusion, researchers reevaluated articles and came to an agreement following arbitration.  Figure 1 illustrates the article selection process.

## 3. Results

The searches extracted 1940 peer-reviewed articles from both electronic databases. Researchers removed any duplicates leaving 1932 journal citations. Six studies adhered to the inclusion criteria producing a small but heterogeneous range of samples and methodologies [16–21]. The results showed that four studies (67%) included participants with various levels of ID [16–19] with the two remaining studies (33%) featuring participants only with Down syndrome (DS) [20, 21]. Temple et al.'s [17] research included individuals with DS and Duane syndrome under the generic term ID. Two additional studies included participants only with severe disabilities [16, 19] while another contained a comparison group without disabilities [21].  Table 1 provides a summary of all studies included under the criteria laid out in the method section and documents author information, key research questions, subjects, design, and measurement tools as well as outcomes.

Four of the studies that met our inclusion criteria attempted to cross validate anthropometric measurements using a criterion measure, namely, ADP [20, 21], DXA [17], and isotope dilution [16]. The validity of skinfold thickness generalized prediction equations was tested in four studies (67%). The prediction equations featured in the included studies were those of Jackson & Pollock [22], Kelly et al. [23], Lohman [24], Jackson et al. [25], Durnin & Womersley [26], Gurka et al. [27], Pencharz & Azcue [28], Slaughter et al. [29], Johnston et al. [30], and Brook [31]. Studies focusing on individuals with DS [20, 21] and severe neurological impairment [16] suggest that further work is needed to validate prediction equations for these specific populations. Results also indicated that skinfold measures may produce high levels of noncompliance amongst different populations with ID [16, 18, 19]. Criterion measures ADP and DXA reported high levels of compliance in individuals with DS [20, 21] and ID [17], respectively.

The feasibility of anthropometric girth measurements (AGM), BMI [17–19], waist circumference [18, 19], tibia length [19], and BIA [16, 18] as measures of body composition were also examined by various researchers in diverse populations with ID. BIA [16, 18] and BMI [17–19] were found to be practical measures for different populations with ID but no such data was available for individuals with DS.

## 4. Discussion

The purpose of this study was to review the validity and reliability of methods used to measure body composition in individuals with ID. Only six studies met the inclusion criteria so it remains difficult to draw definitive conclusions based on such limited data but findings thus far indicate that BMI [17], waist circumference [21], and tibia length measurements [19] may be used reliably on individuals with ID. However, results throw into question the use of skinfold thickness and non population-specific equations on populations with ID including DS [16, 18, 20, 21].

 It is disconcerting that few studies have made valid and reliable measures for assessing body composition amongst individuals with ID especially when one considers elevated levels of obesity and an increasing number of physical activity- and nutrition-based interventions that focus on this population. Included studies contained heterogeneous samples despite the existence of large differences in body composition and fat distribution between participants with different types of disabilities. Only two studies concentrated solely on individuals with DS [20, 21] and, unlike for the general population, no study was gender- or race-specific. Moreover, three studies included samples of participants, which might have included many different subtypes of ID and developmental disabilities such as DS, Duane syndrome, and autism spectrum disorders. Future research may need to ponder further the physiological differences associated with each specific disability.

Two studies indicate that BMI may be a feasible method for assessing body composition in individuals with ID [17, 18]. BMI showed good agreement with DXA and provides a relatively straightforward means of gauging body composition. However, BMI should still be used cautiously as it takes body fat and fat-free mass as one value [32] while Temple et al. [17] also observed that the measure may misclassify some individuals who are obese but these results should be interpreted cautiously as the sample included 17 participants with DS whose fat distribution may be more truncal compared to other disabilities [20]. Waist circumference measurement was found to be feasible in two reports [18, 19], but overall the sensitivity in identifying obesity-related risk factors may vary based on specific populations with ID [33]. 

 One of the main findings of this review was that preexisting prediction equations used on people without disabilities may not be suitable for individuals with ID who possess unique body proportions and characteristics [15–21]. Only two equations were recommended for people with ID across the six studies [28, 29]. Gonzalez-Aguero et al. [20] found that the equation of Slaughter et al. [29] may be acceptable for individuals with DS despite the large limits of agreement. Rieken et al. [16], examining a sample with severe neurological impairment and ID, devised a new BIA-based prediction equation and found it to be more accurate at assessing health status in this specific population than preexisting measures of skinfold thickness. Gonzalez-Aguero et al. [20] found that three additional equations under- or overestimated body fat compared to the reference method ADP [26, 30, 31] while Usera et al. [21] discovered that three prediction equations lacked validity when assessing body composition among young people with DS [22–24]. These findings are disconcerting as many researchers have used the above equations to judge the effectiveness of their health promotion interventions on participants with ID [12].

Until more population-specific equations are introduced, it may be advisable for researchers and practitioners to bypass field measures such as BIA and skinfold in favour of more complex and precise tools [12]. Hydrostatic weighing is used frequently on the general population but may be difficult for individuals with ID as it requires complete submersion underwater; therefore, participant compliance may be difficult to achieve [34]. Usera et al. [21] found ADP to be a convenient alternative for individuals with and without DS and this method has previously shown high reliability and validity in adults when compared to hydrostatic weighing [35]. It is important to state that hydrostatic weighing and ADP are densitometric techniques and therefore, fat mass calculation using these techniques is obtained by assuming that fat-free mass density is relatively stable (at 1.1 kg/L), a cornerstone constant when using a two-compartment model. Temple et al. [17] chose DXA as a reference method and DXA scans have been applied frequently to examine children and adolescents without ID in both clinical and research settings [9, 36]. DXA's potential benefits include its quick scan time and its accurate measurements in diverse populations. DXA displays minimal bias based on age, sex, physical activity level, race, or proportion of body fat [37, 38] and remains relatively straightforward to operate without the need for active participant involvement, which is an important consideration when working with individuals with ID who may not always comply with more invasive measures. DXA can be considered a three-compartment model, thus reducing the variability of assuming a constant fat-free mass composition of two-compartment models. Still, the use of a four-compartment model for developing and/or validating equations for people with ID is absent and is required. The four-compartment models are the state-of-the art methods for assessing fat mass as no assumptions are needed with respect to fat-free mass composition and density which is important in ID individuals as these components can vary significantly from the healthy adult, specifically total body water and mineral.

*Study Limitations*. Several limitations should be considered when interpreting results of this scoping review. The limited number of studies meeting our inclusion criteria often featured small and heterogeneous samples along with quasi-experimental designs, so it may be difficult to generalize results to larger populations with ID. This scoping review represented a preliminary assessment of the potential size and scope of the available research literature in this area and did not include a formal quality assessment. Nonetheless, this review may lay the groundwork for a systematic review in the future and has uncovered several important findings that may require greater attention. 

## 5. Conclusions

Limited research has assessed the validity and reliability of body composition measures for individuals with ID. The current literature contains too few well-conducted studies to evaluate the effectiveness of body composition measures on this population. BMI and waist circumference do remain practical options for professionals working with individuals who have ID. Yet, our review has also revealed that current prediction equations, used with skinfold thickness measurements and BIA, have either underestimated or overestimated body fat when compared to reference methods. Skinfold measurement has also caused compliance difficulties among participants, which calls into question its usefulness in evaluating the body composition. Future research with larger and more homogeneous samples may well be needed in order to uncover alternative methods that provide accurate measurements for such unique populations. There is also a need to place greater emphasis on finding population-specific prediction equations that are suitable for individuals with ID.

## Figures and Tables

**Table 1:** Reliability and validity of body composition measurements in individuals with intellectual disabilities.

Author(s)	Subjects mean (SD)	Measure	Regression equation	Reference method	Results: reliability and validity	Summary
Usera et al. (2005) [21]	14 adults with DS; 38(11) yrs.	SKF AGM	SKF: Jackson and Pollock [22]. AGM: Kelly et al. [23]; Lohman [24]	ADP with BOD POD	Validity: correlations with reference method and RMSE: Jackson et al. (*r* = .54), RMSE = 14.90; Lohman (*r* = .43), RMSE = 13.20; Kelly and Rimmer (*r* = .11), RMSE = 9.82.	Lack of validity in 3-field-based methods. New equations for DS recommended
Verstraelen et al. (2009) [18]	76 adults with ID; 19–72 yrs.	BMI WC BIA SKF	Jackson et al. [25]; Durnin and Womersley [26]	—	Reliability: *Cohen's* kappa with 90% CI. Intertest agreement among BMI & WC (0.61). Agreements between BMI SKF & FFM index, WC to SKF & FFM and SKF to FFM (<0.6)	BIA & WC feasible measures. Lack of reliability and large noncompliance for both SKF (*n* = 5) and FFMI (BIA) (*n* = 14).
Waninge et al. (2009) [19]	45 severe ID; 38(11) yrs.	BMI WC SKF Tibia length		—	Reliability: ICC for all variables (95% CI) except SKF (>0.90).	Measuring tibia length possible. Noncompliance and low reliability noted for all SKF measurements
Temple et al. (2010) [17]	46 adults mild to mod ID; 19–60 yrs.	BMI	—	DXA	Validity: BMI accounted for 68% of variance in %BF (*r* ^2^). Partial correlation of BMI with fat (*r* = 0.91) and fat-free mass (*r* = .12)	BMI reasonable indicator of adiposity.
Rieken et al. (2011) [16]	61 children w/neurol. disability and severe ID; 10(4) yrs.	SKF BIA Tibia length	SKF: Gurka et al.[27]; Rieken et al. [16] BIA: Pencharz and Azcue [28]; Rieken et al. [16]	Isotope dilution	Validity: ICC SKF-Gurka et al. [27] mean Difference = −9.2 ± 16.7; ICC = 0.51; SEE = 5.1 kg; *R* ^2^ = 0.27; Rieken et al. ICC = 0.59; SEE = 7.6 kg; *R* ^2^ =0.44; SEE = 2.2 kg; *R* ^2^ = 0.88; BIA- Pencharz and Azcue [28] Mean difference = 2.6 ± 4.4; ICC = 0.94; Rieken et al. ICC = 0.96; SEE 1.7 kg; *R* ^2^ = 0.92	SKF met with noncompliance (*n* = 12). Low reliability and validity for SKF compared to BIA.
Gonzalez-Aguero et al. (2011) [20]	28 children with DS; 10–20 yrs.	SKF	Slaughter et al. [29]; Durnin and Womersley [26]; Johnston et al. [30]; Brook [31]	ADP	Validity: Slaughter et al. [29] (*r* = 0.105 (*P* = 0.583)); mean difference = 0.69; 95% CI = 25.8; Durnin and Womersley [26] (*r* = 0.529 (*P* < 0.05)); mean difference = 2.34; 95% CI = 18.0; Johnston et al. [30] (*r* = 0.665 (*P* < 0.05)); mean difference = 2.73; 95% CI = 19.6; Brook (*r* = 0.389 (*P* < 0.05)); mean difference = −2.45; 95% CI = 22.3	Slaughter's equation most accurate despite wide LOA. Other equations displayed substantial intermethods difference and under- or overestimation of % BF.

ID: intellectual disability; DS: Down syndrome; AGM: anthropometric girth measurements; SKF: skinfold; BIA: bioelectrical impedance analysis, ADP: air displacement plethysmography; WC: waist circumference; FFMI (BIA): fat-free mass index derived by bioelectrical impedance analysis; %BF: percent body fat; ICC: intraclass correlation coefficients; CI: confidence interval; *r*: coefficient of correlation; LOA: limits of agreement.
